# Editorial: Case reports in pediatric critical care 2022

**DOI:** 10.3389/fped.2023.1176704

**Published:** 2023-03-17

**Authors:** Ryan J. Stark, Dinçer Yildizdas

**Affiliations:** ^1^ Department of Pediatrics, Division of Pediatric Critical Care, Vanderbilt University Medical Center, Nashville, TN, United States; ^2^ Department of Pediatrics, Division of Pediatric Critical Care, Çukurova University School of Medicine, Adana, Türkiye

**Keywords:** pediatric critical care, sepsis, circulation, case reports, technology

**Editorial on the Research Topic** Case reports in pediatric critical care 2022

While critical illness in children has existed since the dawn of humankind, the ability to understand these illnesses and provide therapies in dire circumstances is relatively new. The fledgling field began with the technological advances of the mid-20th century, highlighted by the poliovirus epidemic and the development of “iron lungs” along with the discovery and refinement of antibiotics (1, 2). Further progress gave rise to new devices and surgical advancements designed to improve the respiratory and cardiovascular physiology of even the smallest of humans (3, 4). Additional technological enhancements along with their more widespread use led to the need to cohort these vulnerable children under the care of medical specialists trained to apply physiologic and technical knowledge (5). With this progression, pediatric critical care was born. In the half-century since these advances, the care of critically ill children has progressed tremendously. This progression is highlighted by the research topic “Case Reports in Pediatric Critical Care 2022” in Frontiers in Pediatrics where the cases presented show how pediatric intensivists still encounter challenges and discover new physiologic alterations in caring for the sickest of children.

The host response to an infectious challenge is a fundamental response for survival and its origins can be traced to the most rudimentary organisms. Occasionally, the host response is either insufficient or aberrant leading to severe illness. Despite advances in antimicrobials and preventative treatment, children remain susceptible to pathogens and the consequences of a dysregulated host response. In the most extreme circumstances, extracorporeal technologies are required to support children through their illnesses. Such was in the case by Thery et al. where a child who ingested food contaminated with *Bacillus cereus* developed multiple organ dysfunction. Decontamination with antibiotics reduced the bacterial burden with further toxin reduction through hemofiltration. In total, a favorable outcome was achieved. The use of hemofiltration was similarly applied in a different case of a systemic inflammatory response. In the case by Phan et al. the patient suffered from a second episode of SARS-CoV-2 infection resulting in myocarditis that required extracorporeal therapies. To reduce the inflammatory process, a hemofilter was added to the extracorporeal circuit to remove any circulating cytokines or other pro-inflammatory mediators that could be driving the cardiac dysfunction. The initiation of hemofiltration provided a temporal improvement in the patient's physiological status. Though the concept of blood filtration and purification of disease-inducing mediators is not new, these cases highlight that strategic use of evolving technologies can be successfully utilized to rescue children from severe infectious diseases (6).

Technology has also been used to give us greater insight into the pathophysiology of the diseases encountered in pediatric critical care medicine. In one case by Bottari et al. a young child with SARS-CoV-2-related pneumonia who required mechanical support underwent an assessment of their microcirculation using a handheld device that relies on incident dark field microscopy. The use of this device has the potential to delineate alterations in regional blood flow as well as provide real-time assessment of capillary hemostasis and leukocyte-endothelial interactions. Similarly, another report by Wijers et al. examined a series of pediatric patients with septic shock and determined the vascular responses throughout their hospitalization. Vascular assessments were done utilizing laser doppler perfusion monitoring coupled with pharmaceutical-targeted iontophoresis to determine if endothelial vs. direct smooth muscle function was associated with the overall clinical status. While there are no specific therapies that can target leukocytes or the endothelium during severe infections, future utilization of such techniques described in these reports may allow investigators to examine how new treatments impact these crucial regulators of physiology (7).

Beyond infection-related processes, another commonly encountered pathophysiology in pediatric critical care medicine is circulatory alterations related to the treatment of congenital heart disease. In a case by Hayashi et al. a patient was noted to be hemoconcentrated following surgical correction of tetralogy of Fallot with an aortopulmonary shunt. The use of indirect calorimetry to assess gas exchange helped the treating intensivists realize the shunt was being compromised by the hemoconcentration. This led to a partial exchange transfusion that improved blood flow through the shunt. In a different case by Martin et al. a patient who underwent Fontan palliation for hypoplastic left heart syndrome had a complicated course leading to the need for chronic hemodialysis due to oliguria. While the patient was able to be discharged, complications from the dialysis catheter led to the need to transition to long-term peritoneal dialysis. Despite the concerns about intraabdominal pressure on the passive pulmonary blood flow of the Fontan circuit, the patient ultimately tolerated the transition and did well. Though surgical outcomes after the correction of congenital heart lesions have dramatically improved over the preceding decades, vigilance and consideration of complex physiology are still required to navigate the post-operative complications that may arise (8).

Compromised blood flow to the brain is another challenge that intensivists encounter regularly in practice. In one case by Rizzati et al. the cause of impaired blood flow was due to transient vasoconstriction and vasodilatation of the cerebral arteries inducing infarcts. Treatment involved the application of an intra-arterial calcium channel blocker and phosphodiesterase inhibitor to treat the changing arterial constriction. Though the patient had residual defects from the ischemic episodes, they were able to be discharged to a rehabilitation facility. However, not all patients have a favorable outcome. In a report by Hayashi et al. after a prolonged cardiopulmonary resuscitation event, the patient ultimately developed brain death. This progression was accompanied by alterations in heart rate variability and reductions in the hypothalamic-pituitary-adrenal axis that provided adjunctive information that brain death had occurred. Certainly, optimization of cerebral blood flow is the primary goal in the critically ill child with neurologic injury. But understanding and prognosticating what can be reversed and what cannot is a vital role of pediatric intensivists in caring not only for children but their families as well (9).

In summation, the cases described in this research topic highlight how pediatric intensivists encounter diverse pathology in daily practice. Yet despite children presenting in the most extreme of physiological disturbances, global outcomes in pediatric critical care have improved. In doing so, pediatric intensivists will have to adapt, understanding not only the pathophysiology at the bedside in front of them but the sequelae that critical illness can have once they leave the hospital setting (10). In this regard, there will likely be continued challenges and subsequent advancements in pediatric critical care as the practice of taking care of the sickest of children continues to evolve.
